# Multifaceted Roles of Retromer in EGFR Trafficking and Signaling Activation

**DOI:** 10.3390/cells11213358

**Published:** 2022-10-25

**Authors:** Zhe Yang, Zhengyang Feng, Zebin Li, Rohan D. Teasdale

**Affiliations:** School of Biomedical Sciences, Faculty of Medicine, The University of Queensland, Brisbane, QLD 4072, Australia

**Keywords:** retromer, endosomes, protein trafficking, EGF receptor

## Abstract

Mammalian retromer complex contributes to multiple early endosome-associated trafficking pathways whose origins are dependent on which sorting nexin (SNX) they are complexed with. In an attempt to dissect out the contribution of individual retromer–SNX complexes, we examined the trafficking of EGFR in detail within a series of KO cell line models. We demonstrated that the depletion of retromer subunit Vps35 leads to decreased EGFR protein levels in resting cells with enhanced association of EGFR with lysosomal compartments. Compared to control cells, the addition of EGF to Vps35 KO cells resulted in a reduced rate of EGFR degradation; AKT activation and cell prolferation rates were elevated, while ERK activation remained relatively unchanged. These observations are consistent with a prolonged temporal association of EGFR within early endosomes due to the inefficiency of early endosome-associated protein trafficking pathways or organelle maturation due to retromer absence. We did not fully delineate the discrete contributions from retromer-associated SNXs to the phenotypes observed from retromer Vps35 depletion. While each of the knock-outs of SNX1/2, SNX3, or SNX27 promotes the enhanced association of EGFR with early endosomal compartments, only the decreased EGF-mediated EGFR degradation was observed in SNX1/2 dKO cells, while the enhanced AKT activation was only increased in SNX3 KO or SNX27 KO cells. Despite this, each of the knock-outs showed increased EGF-stimulated cell proliferation rates.

## 1. Introduction

The epidermal growth factor receptor (EGFR) belongs to receptor tyrosine kinase, which is important for cell proliferation, cell survival, development, and tissue regeneration upon the activation of its downstream signaling cascades [1]. EGFR mutations and overactivated EGFR signaling pathways are frequently associated with several types of cancers, including lung, head and neck, and colon cancer [1]. Hence, anticancer therapeutics targeting EGFR function are currently being developed. EGFR trafficking and EGFR signaling activation are highly coordinated but complex processes. EGF promotes the internalization of EGFR in both clathrin-mediated and nonclathrin-mediated endocytosis [2]. Internalized EGFR reaches to the early endosomal compartments, where it can either be sorted into recycling endosomes or ubiquitinated provided the sorting signal traffics it into the multivesicular body (MVB)/lysosomal compartments for the degradative route. In addition, EGFR can also traffic to the nucleus or the trans-Golgi network (TGN) [2,3]. The activation of downstream signaling cascades can trigger from multiple cellular compartments. For example, APPL1 positive early endosome compartments are important for controlling AKT activation at the endosome and dictating its substrate specificities [3], whereas ERK activation can be elicited through the late endosomal compartment [4]. Importantly, the endocytosed EGFR at the endosomes is sufficient to control the activation of these downstream signaling pathways in a spatial and temporal manner [5,6,7].

The evolutionally conserved retromer protein complex contains a core trimer subcomplex composed of Vps26, Vps29, and Vps35 subunits [8]. When associated with different PX domain containing sorting nexin (SNXs) modules, a key protein family for endosomal trafficking, retromer can mediate protein sorting to different endocytic pathways [8,9,10]. Its association with SNX3, containing PX domain only, is required for the recruitment of the retromer core subcomplex to the endosomal membrane but also mediates cargo trafficking [11,12,13]. Moreover, retromer with SNX3 specifically regulates CI-M6PR retrograde trafficking from the early endosomes, which is tethered by GRIP and the coiled-coil domain containing 88 kDa (GCC88) at the TGN [14]. The BAR domain containing SNXs in addition to the PX domain, including SNX1, SNX2, SNX5, and SNX6, can homo- or heterodimerize to promote the endosomal tubule formation, leading to protein retrograde trafficking from the early endosome to the TGN [9]. Furthermore, these SNX-BAR proteins can also directly interact with cargo protein for its sorting [15,16,17]. In contrast to SNX3 or SNX-BAR proteins, when in the complex with SNX27, which also contains a PDZ domain and a FERM domain, both of which are involved in cargo binding, retromer can mediate the cargo trafficking from the early endosomes to the cell surface [18,19,20,21]. Numerous cargo proteins, including several receptor tyrosine kinases, such as c-Met, for retromer have been discovered [21,22]. Some experimental evidence indicates the links between SNXs and EGFR. For example, the interactions of SNX1, SNX3, or SNX5 with EGFR have been observed by various experimental approaches, such as yeast two hybrid study, coimmunoprecipitation and proximity biotinylation labeling [23,24,25]. Furthermore, these studies have utilized either overexpression or siRNA-based knock-down approaches to determine their roles in EGFR. For example, the knock-down of SNX1 increased EGF-induced EGFR degradation in human gefitinib-resistant lung cancer cell line [26], yet no effect on EGF-mediated EGFR degradation was reported upon the knock-down of SNX1 and SNX2 [27]. On the other hand, both SNX5 overexpression and knock-down have been reported to inhibit EGF-induced EGFR degradation [25,28]. Therefore, despite some evidence showing the regulatory roles of SNXs with EGFR, the exact functions that retromer and its associated SNXs have in EGFR trafficking and signaling cascades remain inconclusive. In this study, we identified the multifunctional roles for retromer in EGFR trafficking and EGFR signaling activation. Retromer Vps35 knock-out (KO), SNX1/2 double knock-out (dKO), SNX3 KO, and SNX27 KO cell lines displayed the increased EGFR association with early endosomes, yet only Vps35 KO and SNX1/2 dKO increased the association of EGFR with lysosomes but decreased EGF-mediated EGFR degradation. Moreover, retromer Vps35 KO, SNX1/2 dKO, SNX3 KO, and SNX27 KO cell lines caused either sustained or transient increases on EGF-induced EGFR signaling activation, which correlated with the increased EGF-stimulated cell proliferation rates.

## 2. Materials and Methods

### 2.1. Chemicals and Antibodies

All chemicals were purchased from Sigma-Aldrich (North Ryde, NSW, Australia), except for recombinant human EGF (Catalog: PHG0311) obtained from Thermo Fisher Scientific (Mulgrave, VIC, Australia). Mouse monoclonal antibody against human SNX27 (Catalog: 77799) was purchased from Abcam (Melbourne, VIC, Australia). Mouse monoclonal antibody against β-tubulin (Catalog: T9026) was purchased from Sigma-Aldrich. Mouse monoclonal antibodies to EEA1 (Catalog: 610457), LAMP1 (Catalog: 555798), SNX1 (Catalog: 611483), and SNX2 (Catalog: 611309) were purchased from BD (Franklin Lakes, NJ, USA); Rabbit polyclonal antibody to SNX3 (Catalog: ab56078) was purchased from Abcam. Goat polyclonal antibody to Vps35 (Catalog: NB100-1397) was obtained from Novus Biologicals. Rabbit monoclonal antibodies against EGFR (D38B1) (Catalog: 4267), ASCT2 (D7C12) (Catalog: 8057), phosphor-Ser473 AKT (D9E) (Catalog: 4060), phosphor-Thr202/Tyr204 p44/42 MAPK (D13.14.4E) (Catalog: 4370), mouse monoclonal antibodies against AKT (40D4) (Catalog: 2920), and p44/42 MAPK (3A7) (Catalog: 9107) were purchased from Cell Signaling Technology (CST) (Danvers, MA, USA). Mouse monoclonal antibody against EGFR (Catalog: sc-120) was obtained from Santa Cruz Biotechnology (Dallas, TX, USA). HRP conjugated goat antimouse and goat antirabbit secondary antibodies were purchased from Thermo Fisher Scientific. IRdye 680 and IRdye 800 conjugated fluorescence secondary antibodies were purchased from LI-COR Biosciences (Lincoln, NE, USA). All other Alexa Fluor conjugated fluorescence secondary antibodies were purchased from Thermo Fisher Scientific. 

### 2.2. Cell Culture and Treatments

HeLa control, HeLa Vps35 knock-out (Vps35 KO), HeLa SNX1/SNX2 double knock-out (SNX1/2 dKO), HeLa SNX3 knock-out (SNX3 KO), and HeLa SNX27 knock-out cell line (SNX27 KO) were generated previously as described [14]. All cell lines were cultured in high glucose Dulbecco’s Modified Eagles Medium (DMEM) (Thermo Fisher Scientific), supplemented with 10% Fetal Bovine Serum (FBS), 5 mg/mL penicillin and streptomycin, 2 mM L-glutamine, and maintained in 5% CO_2_ at 37 °C.

For EGF stimulation, HeLa cells were serum starved in high glucose DMEM medium containing 0.2% BSA for 16–18 h before subjected to the stimulation with 100 ng/mL of EGF as indicated. Chloroquine treatment was performed as described previously [29]. In brief, cells were incubated with 50 µM chloroquine in normal DMEM medium for 16 h before subjected to cell harvesting for immunoblotting.

### 2.3. Cell Lysis, SDS-PAGE, and Immunoblotting

Whole cells were lysed in TK lysis buffer containing 50 mM HEPES (pH 7.4), 150 mM NaCl, 1% Triton x-100, and protease inhibitor cocktails as described previously [30].

SDS-PAGE and immunoblotting were performed according to procedures described in previous studies [30]. Protein concentrations in each cell lysate were quantified by bicinchoninic acid (BCA) assay (Thermo Fisher Scientific). Equivalent amounts of protein per sample were resolved on SDS-PAGE gel, and proteins were transferred onto Immobilon-PDVF membranes (Merck Millipore, Burlington, MA, USA) using Trans-Blot SD semidry transfer cell system (Bio-Rad, South Granville, NSW, Australia). After completion of transfer, the membranes were incubated with diluted primary antibodies overnight at 4 degrees, before processing to detect signals either by HRP-conjugated secondary antibodies using Clarity Western ECL Substrate kit (Bio-Rad) on ChemiDoc MP System (Bio-Rad) or by IRdye-conjugated fluorescence secondary antibodies using Li-COR Odyssey Infrared Imaging System (Millennium Science, Mulgrave, VIC, Australia). Membrane analysis and quantification were conducted using Image J version 1.53s (NIH, Bethesda, MD, USA) or Odyssey Imaging software version 3.1 (Li-COR, Lincoln, NE, USA).

### 2.4. Cell Proliferation Assay

Cell proliferation was measured using the MTT assay as described previously [29]. HeLa cells were seeded at 7500 cells in 100 μL of complete DMEM containing L-glutamine and 10% FBS per well in a 96-well plate format. Cells were grown for 48 h before incubation with 20 μL of 5 mg/mL of MTT in PBS for 3.5 h in a 37 °C incubator. Then, the medium was removed, and cells were dissolved in 150 μL of MTT solvent containing 4 mm HCl and 0.1% NP-40 in isopropanol. After 15 min incubation, optical absorbance was measured under 590 nm wavelength using a microplate reader (BioTek Instruments, Winooski, VT, USA).

### 2.5. Determination of Cell Surface EGFR Levels Using Flow Cytometry

Cell surface EGFR levels were determined by flow cytometry. After treating with serum-starved cells with 100 ug/mL of EGF for various times, cells were washed with ice-cold PBS and dissociated with 1 mM EDTA in PBS. An amount of 1 × 10^6^ cells were pelleted and fixed with 2% paraformaldehyde (PFA) in PBS for 30 min. Cells were then directly incubated with 1 ug of mouse monoclonal anti-EGFR antibody for 30 min, followed by Alexa Fluor 488 conjugated goat antimouse IgG (1:500) for additional 30 min. After extensive washing, cells were resuspended with 1% PFA in PBS for flow cytometry. Flow cytometry data were acquired using BD Fortessa Flow Analyser (BD Biosciences, San Jose, CA, USA) and analyzed on FlowJoTM v10 software (FlowJo LCC, Ashland, OR, USA).

### 2.6. Immunofluorescence Microscopy and Image Analysis

HeLa cells grown on coverslips were fixed and permeabilized in ice-cold methanol for 5 min, indirect fluorescence microscope procedures were processed as described previously [29]. After blocking with 2% BSA in PBS for 30 min, cells were costained with anti-EGFR (1:200) and anti-EEA1 (1:200) or anti-LAMP1 (1:200) primary antibodies for 1 h at room temperature followed by 1 h incubation with Alexa Fluor 488 conjugated donkey antirabbit and Alexa Fluor 555 conjugated donkey antimouse secondary antibodies (Thermo Fisher Scientific). Coverslips were mounted on glass microscope slides using Dako fluorescence mounting medium (Agilent, Santa Clara, CA, USA), and the images were taken under a Leica SP8 DMi8 inverted confocal laser scanning microscope equipped with 63× glycerol (Numerical Aperture, NA = 1.3) objective. Images were processed and colocalization analysis was performed using Fiji Image J (version 1.53s, National Institute of Health, Bethesda, MD, USA) as described previously. Multichannel images were threshold in each channel; the colocalization was quantified using Fiji Image J JACoPplugin and was represented as colocalization Pearson’s correlation coefficient (r).

### 2.7. EGFR Transcript Analysis by RNA-Seq

Triplicated total RNA samples of HeLa control and Vps35 KO cells were extracted using Qiagen RNeasy Mini Kit (Doncaster, VIC, Australia). RNA-Seq library preparation and sequencing were conducted in the Sequencing Facility at the Institute for Molecular Bioscience (IMB). Illumina Strander mRNA Prep Kit (Illumina, Melbourne, VIC, Australia) was used for library preparation, and Illumina NextSeq 500/550 High Output Kit v2.5 (75 Cycles, Illumina) was used for sequencing on Illumina NextSeq 500 system. RNA-Seq data analysis was performed using Galaxy Platform (version 22.01) provided by Galaxy Australia. In brief, quality controls of the reads were performed using FastQC and Cutadapt, then mapped to a reference genome (Hg38, human genome build 38) using STAR [31]. Following, the number of reads for EGFR transcripts (ENSG00000146648) from HeLa control and Vps35 KO cells were counted using FeatureCounts [31].

### 2.8. Statistical Analysis

Statistical analysis and graph generations were performed using GraphPad Prism9 (version 9.3.1, Graphpad Software Inc., San Diego, CA, USA) or RStudio (version 1.4.17, RStudio Team, Boston, MA, USA) with ggplot2 package. Error bars on the graphs were represented as the standard error of mean (±SEM). All *p* values were calculated using a two-tailed student *t*-test. *p* < 0.05 was considered as significant.

## 3. Results

### 3.1. Retromer Depletion Reduces EGFR Levels but Decreases EGF-Induced EGFR Degradation

**Figure 1:**
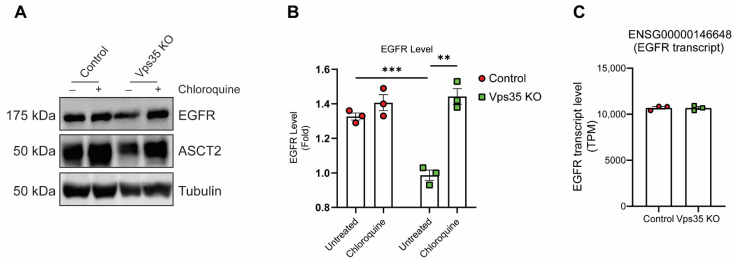
Retromer depletion decreases EGFR levels in HeLa cells. (**A**) Steady-state HeLa control and Vps35 KO cells were treated with 50 µM chloroquine in complete-DMEM media for 16 h. Cells were then harvested and equal amounts of protein samples were subjected for SDS-PAGE and immunoblotting with the antibodies against EGFR and ASCT2, whereas tubulin served as a loading control. Representative blots from three independent experiments are shown. (**B**) The fold differences for EGFR levels are presented (means ± SEM). Two-tailed student’s *t*-test indicated the difference between HeLa control and Vps35 KO cells, *** *p* < 0.001, HeLa control vs. Vps35 KO (untreated); ** *p* < 0.01, Vps35 KO (untreated) vs. Vps35 KO (chloroquine). (**C**) EGFR transcript levels between HeLa control and Vps35 KO cells were measured by RNA-Seq analysis from three replicate samples as represented by Transcripts Per Million (TPM).

To directly examine the functions of the retromer complex in EGFR trafficking, firstly, we compared the total EGFR protein levels in the HeLa control cell line with the retromer Vps35 knock-out (Vps35 KO) cell line by immunoblotting. We observed that the EGFR protein levels within Vps35 KO cells was significantly decreased, when compared to HeLa control (Figure 1A,B). Similarly, ASCT2, a protein established to be recycled via SNX27-retromer, was also decreased in Vps35 KO cells, consistent with previous reports [32]. To block lysosomal degradative pathways, cells were pretreated with chloroquine which, as expected, resulted in increased EGFR and ASCT2 protein levels in Vps35 KO cells (Figure 1A,B). Furthermore, EGFR mRNA transcript levels between HeLa control and Vps35 KO cell were similar (Figure 1C). Overall, this data indicates that within retromer Vps35 knock-out cells EGFR has an altered protein trafficking itinerary resulting in reduced protein levels due to its miss-sorting and degradation.

**Figure 2:**
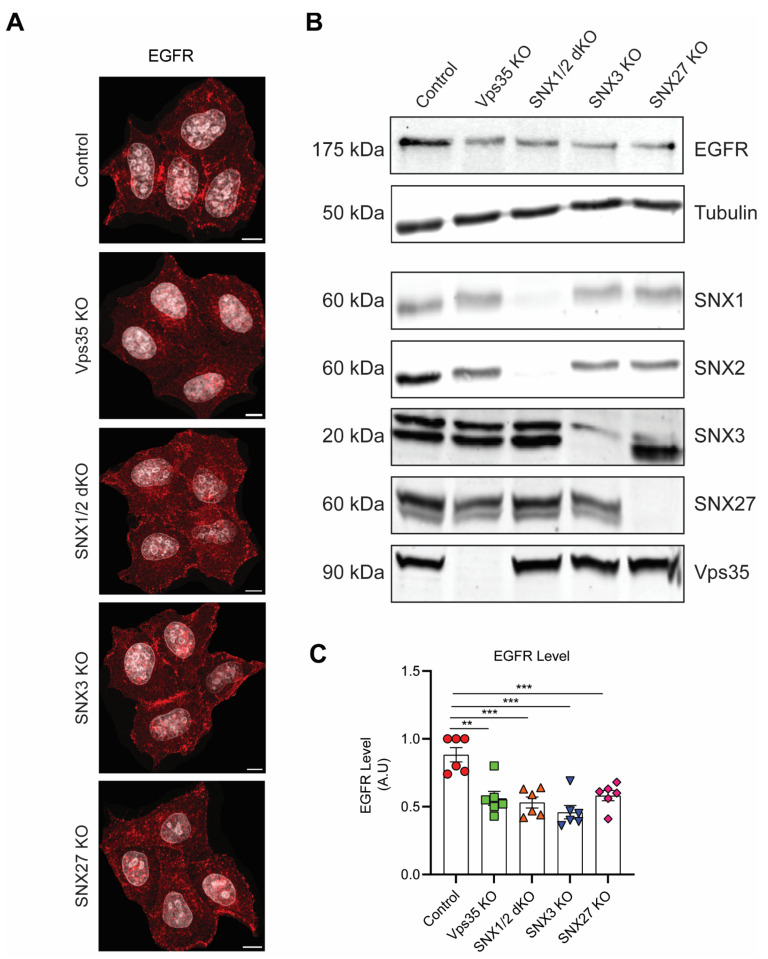
Depletion of retromer or retromer-associated SNXs decreases EGFR protein levels. (**A**) Steady-state HeLa control, Vps35 KO, SNX1/2 dKO, SNX3 KO, and SNX27 KO cell monolayers grown in complete-DMEM media on coverslips were fixed in ice-cold methanol, stained with EGFR antibody, and count-stained with DAPI. Images were captured on a Leica SP8 DMi8 confocal microscope using a 60× glycerol objective (Scale Bar, 10 µm). (**B**) Equal amounts of protein samples from HeLa control, Vps35 KO, SNX1/2 dKO, SNX3 KO, and SNX27 KO cells were subjected to SDS-PAGE and immunoblotting with the antibodies against EGFR, SNX1, SNX2, SNX3, SNX27, and Vps35, whereas tubulin served as a loading control. Representative blots from at least three independent experiments are shown. (**C**) The fold differences for EGFR protein levels are presented (means ± SEM). Two-tailed student’s *t*-test indicated the difference between HeLa control and HeLa KO cells, ** *p* < 0.01, HeLa control vs. Vps35 KO; *** *p* < 0.001, HeLa control vs. SNX1/2 dKO; *** *p* < 0.001, HeLa control vs. SNX3 KO; *** *p* < 0.001, HeLa control vs. SNX27 KO.

To further determine the contributions of each retromer-associated SNXs in EGFR sorting, we compared HeLa control cells with several other HeLa cell lines, in which retromer-associated SNX proteins were depleted, including SNX1/SNX2 double knock-out (SNX1/2 dKO), SNX3 KO, and SNX27 KO. Using indirect fluorescence microscopy, cells exhibited typical cell surface staining when labelled with EGFR antibody in all cell lines under steady-state conditions (Figure 2A). However, when cell lysates were extracted for SDS-PAGE and immunoblotting, immunoblotting demonstrated that the total EGFR levels in Vps35 KO, SNX1/2 dKO, SNX3 KO, and SNX27 KO cells were all significantly reduced when compared to control cells (Figure 2B,C).

**Figure 3:**
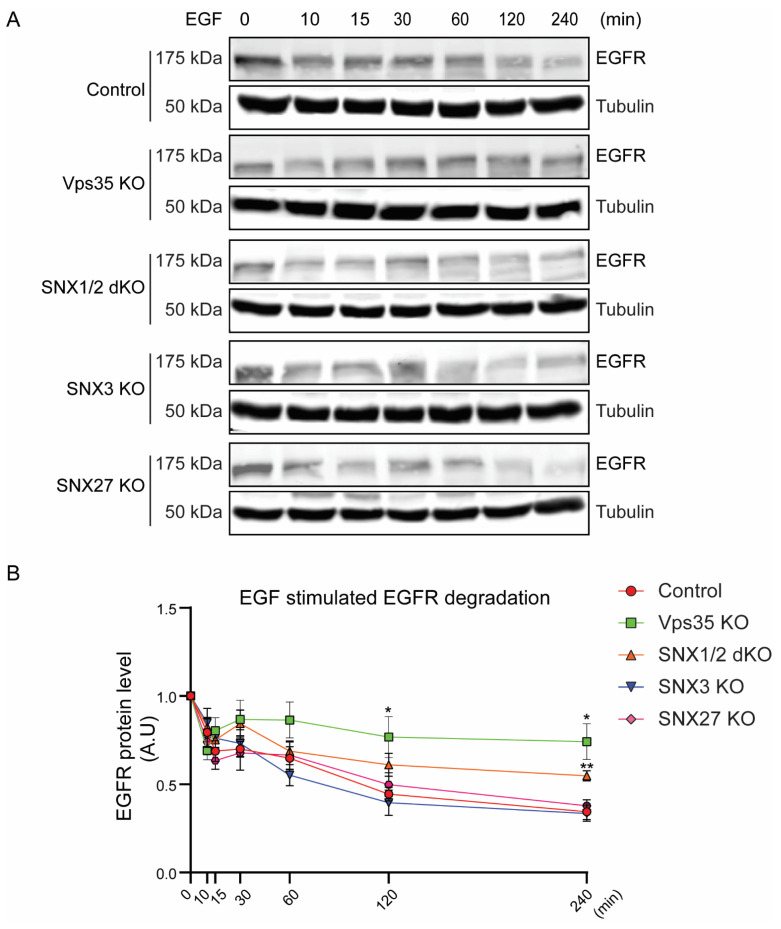
Retromer Vps35 or SNX1/2 depletion altered EGF-stimulated EGFR degradation. (**A**) Serum-starved cells were treated with 100 ug/mL of EGF for different time points. At indicated time points, cells were harvested, and equal amounts of protein samples were used for immunoblotting with EGFR antibody. Representative blots from at least three independent experiments are shown. (**B**) EGFR levels at each time point were normalized to the EGFR level at 0 min for each cell line. Graph represents the difference of EGFR levels during EGF stimulation (means ± SEM). Two-tailed student’s *t*-test indicated the difference, * *p* < 0.05, HeLa control vs. Vps35 KO (120 min); * *p* < 0.05, HeLa control vs. Vps35 KO (240 min); ** *p* < 0.01, HeLa control vs. SNX1/2 dKO (240 min).

High-dose EGF stimulation promotes the sorting and the delivery of EGFR to the lysosomal compartments for its degradation and termination of the associated cell signaling pathways [2]. To investigate if the rates of EGF-stimulated EGFR degradation are differentially regulated between control and retromer KO cell lines, serum-starved HeLa cells were stimulated with 100 ng/mL of EGF for prolonged time points, and cell lysates were extracted for immunoblotting with EGFR antibody (Figure 3). We observed a decrease in the EGFR level after 10 min post-EGF stimulation in the HeLa control cells, similar to other reported studies [33]. We further observed about 50% reductions in the EGFR level after 120 min of EGF stimulation when compared to untreated HeLa control cells, consistent with EGF-induced EGFR degradation. In contrast, the relative EGFR protein levels remained at a higher level in HeLa Vps35 KO cells after both 120 and 240 min of EGF stimulation. In addition, we also observed that the relative EGFR levels in HeLa SNX1/2 dKO cells were higher than control cells after 240 min of EGF stimulation. However, both HeLa SNX3 KO and SNX27 KO cells showed similar EGF-induced degradation kinetics as control. Together, the data suggests that the depletion of retromer core complex as well as SNX1/2-SNX-BAR proteins can restrict EGF-stimulated EGFR delivery to the lysosomal compartments for the degradation.

### 3.2. Retromer Depletion Alters the Kinetics of EGFR Endosomal Trafficking

**Figure 4:**
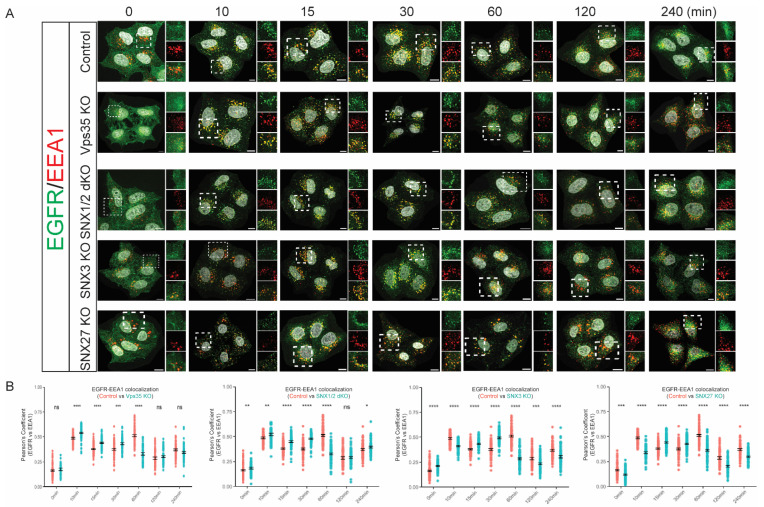
Retromer Vps35 and SNXs depletion altered the kinetics of EGF-stimulated EGFR sorting to early endosomes. (**A**) Serum-starved HeLa control, Vps35 KO, SNX1/2 dKO, SNX3 KO, and SNX27 KO cells were treated with 100 ug/mL of EGF for the time points as indicated before fixation and immunolabeling with EGFR and EEA1 antibodies. Images were captured on a Leica SP8 DMi8 confocal microscope using a 60× glycerol objective (Scale Bar, 10 µm). (**B**). Analysis of colocalization between EGFR and EEA1 is presented by Pearson’s correlation coefficient. The value shows the difference between HeLa control cells and KO cells (means ± SEM). Two-tailed student’s *t*-test indicated the difference between HeLa control and KO cells at the time points as indicated, ns, not significant, * *p* < 0.05; ** *p* < 0.01; *** *p*< 0.001; **** *p* < 0.0001.

We showed that the depletion of retromer Vps35 core subunit and SNX1/2 dimer caused a decrease in EGF-promoted EGFR degradation. Next, we investigated if it could be due to the changes in EGFR endosomal trafficking in these cell lines. Firstly, serum-starved HeLa cell monolayers on coverslips were stimulated with 100 ng/mL of EGF for various time points before being fixed for costaining of EGFR antibody with EEA1 antibody—an early endosome marker, and the degrees of the colocalization between EGFR and EEA1 were analyzed by Pearson’s correlation coefficient (Figure 4). During EGF stimulation, control cells showed a biphasic pattern of the colocalization between EGFR and EEA1, where the highest degrees of colocalization at 10 min and 60 min post-EGF stimulation. The first peak at 10 min could represent newly internalized EGFR entering early endosomal compartments, whereas the second peak at 60 min could represent the recycled EGFR re-entering into the endocytic pathway. Contrary to control cells, the colocalization between EGFR and EEA1 at 10 min, 15 min, and 30 min of EGF stimulation was significantly increased in Vps35 KO cells. Moreover, the second peak for the highest colocalization between EGFR and EEA1 at 60 min of EGF stimulation as in control cells was not observed in Vps35 KO cells. This indicates that retromer Vps35 depletion could promote EGFR retention into early endosomal compartments by prevention of EGFR recycling. Similarly, we found that the SNX1/2 dKO cells displayed similar trends as the Vps35 KO cells for EGFR trafficking through early endosomal compartments. SNX1/2 dKO cells showed the increased colocalization between EGFR and EEA1 after 10 min, 15 min, and 30 min of EGF stimulation (Figure 4). As observed from Vps35 KO cells, the second peak for the colocalization between EGFR and EEA1 at 60 min was also not observed in SNX1/2 dKO cells (Figure 4). Although the association between EGFR and EEA1 at 10 min poststimulation in SNX3 KO and SNX27 KO cells was significantly increased compared with control, the kinetics for EGF-stimulated EGFR colocalization with EEA1 in these cell lines were largely consistent with that observed from Vps35 KO and SNX1/2 dKO cells, where it increased colocalization at 15 min and 30 min but decreased association at 60 min poststimulation (Figure 4). Taken together, our data suggests retromer depletion sustains EGFR sorting to the early endosomal compartments.

**Figure 5:**
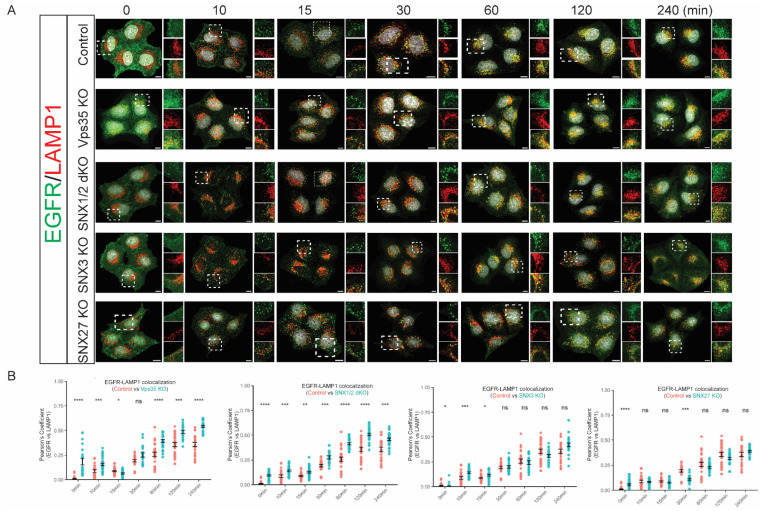
Retromer Vps35 and SNX1/2 depletion altered the kinetics of EGF-stimulated EGFR sorting to lysosomes. (**A**) Serum-starved HeLa control, Vps35 KO, SNX1/2 dKO, SNX3 KO, and SNX27 KO cells were treated with 100 ug/mL of EGF for the time points as indicated before fixation and immunolabeling with EGFR and LAMP1 antibodies. Images were captured on a Leica SP8 DMi8 confocal microscope using a 60× glycerol objective (Scale Bar, 10 µm). (**B**). Analysis of colocalization between EGFR and LAMP1 is presented by Pearson’s correlation coefficient. The value shows the difference between HeLa control cells and KO cells (means ± SEM). Two-tailed student’s *t*-test indicated the difference between HeLa control and KO cells at the time points as indicated, * *p* < 0.05; ** *p* < 0.01; *** *p* < 0.001; **** *p* < 0.0001, ns, not significant.

Next, we examined the kinetics of EGF-stimulated EGFR delivery into lysosomal compartments in these cell lines. EGF-stimulated HeLa cell monolayers were fixed at various time points for costaining of EGFR antibody with LAMP1 antibody, a lysosomal marker, and Pearson’s correlation coefficient was used to measure the degrees of the colocalization between EGFR and LAMP1. HeLa control cells showed a steady increase in colocalization between EGFR and LAMP1 during the EGF stimulation time course (Figure 5), consistent with the sorting of activated EGFR to the lysosomal compartments for the degradation. Serum-starved but untreated Vps35 KO cells had an increased colocalization between EGFR and LAMP1. This is consistent with the reduced total levels of EGFR observed in these cells indicating a higher level of EGFR being constitutively delivered to lysosomes for degradation. Like in the control cells, the colocalization of EGFR to LAMP1 was further significantly enhanced at 60 min, 120 min, and 240 min of EGF stimulation in Vps35 KO cells (Figure 5). This indicates that retromer Vps35 depletion could actively promote EGFR sorting into late endosomal and lysosomal compartments but has an impaired capacity for lysosomal degradation or EGFR recycling. SNX1/2 dKO cells displayed a similar trend as the Vps35 KO cells for the sorting of EGFR to the lysosomal compartments. As measured from the fluorescence microscopy, the SNX1/2 dKO cells showed the significantly increased colocalization between EGFR and LAMP1 during EGF stimulation (Figure 5). On the other hand, unlike the Vps35 KO or SNX1/2 dKO cells, the kinetics of EGF-regulated EGFR sorting into lysosomal compartments in SNX3 KO and SNX27 KO cells were largely similar to control cells (Figure 5), indicating that SNX3 or SNX27 only have minor roles in the sorting of EGFR to the lysosomal compartments.

### 3.3. Retromer Depletion Increases EGF-Stimulated EGFR Signaling and Its Associated Cell Proliferation

**Figure 6:**
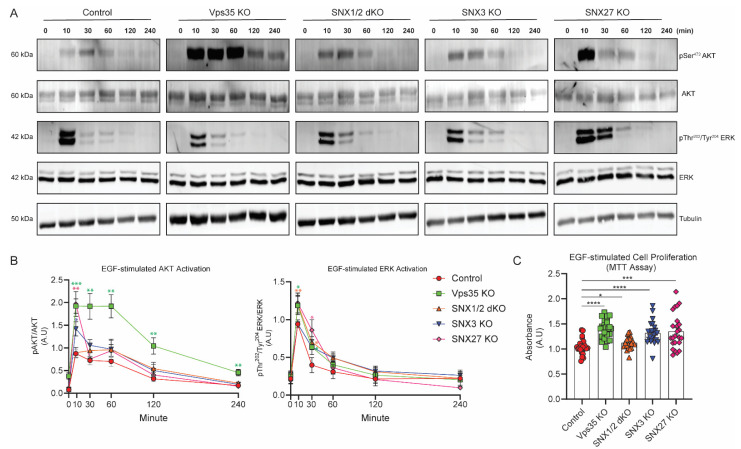
Retromer Vps35 and SNXs depletion altered the kinetics of EGF-stimulated EGFR signaling activation. (**A**) EGF-treated cells were lysed at various time points as indicated. Equal amounts of protein samples were subjected to immunoblotting and labelled with the antibodies against phosphor-Ser473 AKT, AKT, phosphor-Thr202/Tyr204 ERK, ERK, or tubulin. Representative images are from at least three independent experiments. (**B**) Normalizations of phosphor-Ser473 AKT to AKT or phosphor-Thr202/Tyr204 ERK to ERK are represented as the fold difference for AKT and ERK activation levels between HeLa and KO cells (means ± SEM). Two-tailed student’s *t*-test indicated the difference between HeLa and KO cells at the time points indicated, * *p* < 0.05; ** *p* < 0.01; *** *p* < 0.001. (**C**) HeLa control and KO cells were either cultured in serum-free DMEM medium or serum-free DMEM medium containing 100 ng/mL of EGF for 24 h before being subjected to MTT assay. Optical absorbance was measured under 590 nm wavelength using a microplate reader, and the values were calculated from the absorbances under EGF-treated conditions normalized to serum-free conditions, representing the fold difference in cellular proliferation rates (means ± SEM). Two-tailed student’s *t*-test indicated the difference between HeLa and KO cells, * *p* < 0.05, control vs. SNX1/2 dKO; *** *p* < 0.001, control vs. SNX27 KO; **** *p* < 0.0001, control vs. Vps35KO or control vs. SNX3KO.

It is well-established that signaling activation can be derived from the plasma membrane, early endosomal compartments, or late/lysosomal compartments [2,4,34]. Next, we examined if the changed kinetics of EGFR sorting to early endosomal and lysosomal compartments would have consequences on the EGF-mediated downstream of EGFR signaling. To determine this, serum-starved cells were stimulated with 100 μg/mL of EGF for the time points as indicated, and equal amounts of protein samples were used for SDS-PAGE and immunoblotting with phosphor-Ser473 AKT, phosphor-Thr202/Tyr204 ERK, total AKT, and total ERK antibody, whereas beta-tubulin antibody was used as a loading control. AKT activation in the HeLa control as measured by phosphor-Ser473 AKT antibody reached its maximal at 10 min post-EGF stimulation, consistent with previous findings that EGF-mediated AKT activation occurs within early endosomal compartments. The sustained AKT activation was maintained through a 60 min period before being deactivated. AKT activation in Vps35 KO cells followed a similar pattern as control cells but the AKT Ser473 phosphorylation levels were enhanced (Figure 6A,B). In contrast to Vps35 KO cells, EGF-stimulated AKT activation in SNX1/2 dKO, SNX3 KO, and SNX27 KO cells was only transiently enhanced at 10 min or 30 min of EGF treatment (Figure 6A,B). The kinetics and amplitudes of EGF-mediated late endosome-associated ERK activation, as measured by ERK Thr202/Tyr204 phosphorylation, were largely comparable among these cell lines. Since we observed the sustained or transiently increased EGF-stimulated AKT activation in retromer Vps35 KO cells as well as in SNX1/2 dKO, SNX3 KO, and SNX27 KO, we determined the outcomes of the altered downstream of EGFR signaling cascades by measuring EGF-stimulated cell proliferation rates. Cells were incubated with serum-free medium only or serum-free medium containing 100 ng/mL of EGF for 24 h before being subjected to MTT assay (Figure 6C). The data shows that Vps35 KO displayed a 1.35× fold increase in cell proliferation rates while SNX3 KO (1.26× fold) and SNX27 KO (1.29× fold) were also elevated; the SNX1/2 dKO (1.08× fold) proliferation rate was closer to the control cells (Figure 6C).

In summary, our study showed the retromer complex and its association with SNX proteins having functions on EGFR-sorting and EGFR-signaling activations. We provided the evidence that retromer Vps35 and SNX1/2 depletion leads to a reduced ability for EGF-mediated EGFR degradation, despite the increased colocalization of EGFR with the lysosomal compartment. Furthermore, we observed either sustained or transiently increased EGF-stimulated AKT activation in knock-out cells, which could drive the enhanced cell proliferation rates as observed.

## 4. Discussion

In this study, we provided some evidence on the multifaced roles of the retromer complex and its associated SNXs in EGFR trafficking and EGFR signaling. Firstly, we showed that the depletion of retromer Vps35 or SNXs alters the kinetics of EGFR trafficking: (1) the depletion of retromer Vps35, SNX1/2, SNX3, or SNX27 increased EGF-mediated EGFR accumulation within earlier endosomal compartment, yet the increased EGFR accumulation within the lysosomal compartment was only observed upon retromer Vps35 or SNX1/2 dKO depletion; (2) the depletion of Vps35 or SNX1/2 partially decreased EGF-induced EGFR degradation; (3) finally, the depletion of Vps35, SNX1/2, SNX3, or SNX27 sustainably or transiently increased EGF-activated EGFR signaling, leading to increased cell proliferation rates.

Dissecting molecular actions of different endosomal components in EGFR trafficking and EGFR signaling have been widely studied, including retromer and SNXs. However, due to different experimental approaches, such as cell types, overexpression, transient knock-down, or endogenous knock-out, results are often inconsistent. Despite proteomics studies uncovering many cargo proteins, including receptor tyrosine kinases, such as IGFR1, c-MET, whose trafficking can be mediated directly by retromer or SNXs, only limited evidence shows that retromer or SNXs can directly bind with EGFR. A previous yeast two-hybrid study showed that SNX1 could bind to EGFR in the region containing amino acid (aa) from 943 to 957 [24]. A peptide array screen also suggests SNX27′s FERM domain could interact with phosphorylated NPXY motif within EGFR and other receptor tyrosine kinases [35]. Recently, SNX3 was also shown to bind with EGFR using biotin proximal labelling approach, and SNX5 immunoprecipitated with EGFR in Huh7—a hepatocyte-derived carcinoma cell line [23,25]. Despite all these, solid evidence for direct interactions between EGFR and retromer/SNXs is still largely lacking.

The steady-state protein levels of EGFR were reduced in Vps35 KO cells. Despite the reduced lysosomal hydrolase activities observed in Vps35 KO cells [14], these changes were not due to impaired lysosomal function. We did not see any accumulation of EGFR in the lysosomes in Vps35 KO cells, and the inhibition of lysosomal degradative pathways in Vps35 KO cells, via chloroquine treatment, reverted the EGFR protein levels to that observed in HeLa control cells. We, therefore, proposed that in the absence of efficient protein trafficking from early endosomes, more internalized EGFR in Vps35 KO cells is retained within these organelles resulting in its increased exposure to protein degradation pathways as maturation occurs. This may or may not involve the post-translational modification of EGFR by ubiquitin which is well-established to regulate its targeted degradation [36,37]. Alternatively, while EGFR protein levels can be altered by transcriptional regulation [38,39], we observed no changes in the EGFR transcription levels in Vps35 KO cells when compared to control cells. However, we could not formally discount the possibility that protein translation rates may be impaired due to the reduced mTORC1 signaling in the absence of Vps35 [40,41].

Upon EGF treatment, we observed increased EGFR within early endosomal compartments in retromer Vps35-, SNX1/2-, SNX3-, or SNX27-depleted cells. It could be due to the increased EGFR internalization rates in depleted cells; however, we only observed more rapid removal of EGFR in Vps35 and SNX3 KO cells after 5 min of EGF stimulation by flow cytometry analysis. Accumulation in the SNX1/2- and SNX27-depleted cells may be due to inefficient recycling to the plasma membrane directly or indirectly via the retrograde pathway. It was still unclear if retromer depletion alters the kinetics of endosomal maturation or phosphoinositide metabolism, thereby affecting EGFR trafficking at the level of the early endosome. For example, the increased PI(3)P levels could cause the accumulation of early endosomes to disrupt EGFR trafficking [42]. Retromer requires Rab7 GTPase for its endosomal recruitment [12,43]. In contrast, retromer Vps35 depletion can either cause a decrease in TBC1D5—a Rab GAP for Rab7 or alter Rab7 distribution, overall affecting Rab7 activity and the late endosome maturation process [44]. Nevertheless, the increased EGFR association with the lysosomal compartment and decreased EGF-stimulated EGFR degradation in Vps35 KO was consistent with previous observations that these cells have reduced proteolytic activities and altered morphologies consistent with a defect with the cyclic lysosome maturation process [14]. The decreased EGFR degradation observed from SNX1/2 dKO cells could be explained by their role in lysosomal maturation [45]. Hepatocyte growth factor-regulated tyrosine kinase substrate (Hrs)—a key component for the endosomal sorting complex required for transport (ESCRT)-0—is critical to mediate the sorting of ubiquitinated EGFR into multivesicular bodies (MVBs) for the lysosomal degradation. However, Hrs overexpression has been shown to prevent EGF-mediated EGFR degradation, which could be due to the competition between Hrs and SNX1 for EGFR binding, despite SNX1 being able to directly interact with Hrs [46]. Our data was consistent with the model that SNX1 can function in ligand-activated EGFR sorting to lysosomal compartment for the degradation. In addition to SNX1 and SNX2 interactions, SNX1 also directly interacts with SNX5 to form the heterodimer of SNX-BAR for endosomal tubule formation and mediates cargo retrograde trafficking [15,16,17]. Within the SNX1/2 dKO cells, the protein levels of both SNX5 and SNX6 were significantly reduced mostly due to instability when unable to form heterodimers [14]. Consistent with our observation on the positive roles for SNX1/2 in the sorting of EGFR into degradative pathway, a previous study also displayed a similar role for SNX5. The interaction of SNX5 with type I gamma phosphatidylinositol phosphate 5-kinase i5 (PIPKIγi5), an enzyme for PtdIns4,5P2 synthesizing, modulates the delivery of EGFR into MVB for the degradative pathway [28]. Either SNX5 or PIPKIγi5 inhibition by siRNA knock-down decreased EGFR binding to Hrs, thereby preventing it from EGFR degradation [28]. However, a negative role of SNX5 in EGFR degradation has also been suggested, as SNX5 overexpression decreased EGF-stimulated EGFR degradation [25]. Hence, the threshold levels of endosomal sorting machineries, including SNXs, are required for balancing EGFR trafficking, and further experiments are required to confirm this model.

Once the ligand binds to EGFR, phosphorylated EGFR becomes activated to induce the activation of EGFR downstream signaling from various subcellular compartments, including cell surface, early endosomal compartments, late endosomal/lysosomal compartments, and nucleus [2]. In the case of AKT activation, it can be activated on the plasma membrane. Moreover, it was also shown that AKT activation can occur in the early endosomes, particularly arising from the subpopulation of early endosomes that is APPL1-positive, which dictates AKT substrate specificities [34]. Furthermore, the internalized EGFR in the endosomes is sufficient to transduce downstream signaling activation [5,7]. Consistent with the increased EGFR localization to EEA1-positive early endosomes in Vps35-depleted cells, we observed a sustainably increased AKT activation compared to control cells. Similar findings were also observed in other models. For example, Vps35 loss of functions promotes osteoclastogenesis through receptor activator of NF-kB (RANK) ligand (RANKL)-induced RANKL signaling, including AKT activation [47]. In addition to the increased EGFR localization to early endosomes in Vps35-depleted cells, other possibilities could also explain the observed enhanced AKT activation. Retromer depletion causes the increased RAB7 GTPase activity due to the decreased TBC1D5 level, which has GAP activity towards RAB7 [41]. This imbalanced RAB7 activity disrupts amino-acid-stimulated mTORC1 (mTOR/Raptor) signaling, it is possible that the increased mTORC2 (mTOR/Rictor) activity by unknown mechanisms acts as the upstream kinase for AKT activation [41]. For example, the distribution of lysosomes within the cells can mediate mTORC2 and AKT signaling [48]. Indeed, we also observed the transiently increased AKT activation in SNX27-depleted cells. A previous proteome study reported that SNX27 can interact with mTOR and Rictor directly [21]. Hence, it is conceivable that SNX27 may act as a negative regulator for mTORC2 which prevents the recruitment of mTORC2 to the plasma membrane for AKT activation. Moreover, PH domain and Leucine rich repeat Protein Phosphatase 1 (PHLPP1) and PHLPP2 are protein phosphatases for protein dephosphorylation, including AKT phosphorylation. Both PHLPP1 and PHLPP2 contain the PDZ domain binding motif (PDZbm) at their C-terminal, which can directly interact with the PDZ domain of SNX27. SNX27 depletion, therefore, may reduce the local recruitment of PHLPP1 and PHLPP2 relative to activated AKT preventing its dephosphorylation [21]. Changes in these molecular interactions spatially or temporally may also explain the prolonged AKT activation observed in Vps35 KO, but not in SNX1/2 dKO cells, despite both cells showing the increased association with early endosomes and the decreased EGF-stimulated EGFR degradation. Nevertheless, further studies are required to determine the functions of retromer and SNX27 in EGF-induced mTORC2 and AKT activation. In contrast to AKT activation at the plasma membrane or early endosome, the late endosomal adaptor molecule p14 (LAMTOR2) forms the complex with MAPK/ERK kinase 1 partner (MP1) and ERK at late endosome to activate ligand-induced ERK activation [4]. In addition, manipulations of late endosome localization can delay EGF-induced EGFR degradation, while prolonging ERK activation [5,7]. However, we only observed a modest transient increase in ERK activation in retromer-depleted cells. Furthermore, recent studies also suggest that a small population of cell surface EGFR, rather than endosomal EGFR, is responsible for ERK activation [49]. Therefore, the contributions of endosomal EGFRs in retromer- and SNXs-depleted cells to EGFR signaling need to be fully characterized. Despite retromer Vps35 KO cells leading to prolonged AKT activation, whereas other SNXs KO cells only have transient or modest effects on AKT or ERK activation, the depletion of retromer Vps35 or retromer-associated SNXs all leads to the increase in EGF-mediated cell proliferation. It was shown that EGF mediates cell proliferation through two distinct phases of signaling activation [50]. In this study, we only assessed EGF-stimulated AKT and ERK activation at the relatively early time points, whereas we did not determine AKT and ERK activation during chronic EGF-stimulation conditions. Furthermore, it is known that EGFR activation can also lead to the activation of Janus kinase (JAK) and signal transducer and activator of transcription (STAT) pathways, which in turn binds with several transcription factors resulting in transcriptional changes [51]. It was reported that retromer Vps35 depletion leads to the prolonged association of interferon receptor with the early endosome and the increased STAT1-dependent gene transcription [52]. Hence, it is possible that retromer Vps35 or retromer-associated SNXs depletion may increase EGF-mediated phenotypes via the activation of the JAK-STAT pathway to contribute to the observed increased cell proliferation rates within the KO cells.

## 5. Conclusions

Overall, in this study, we reported the multifaced roles of retromer with SNXs in EGFR trafficking and EGFR signaling, but not the distinct mechanisms/models involved. Firstly, retromer Vps35 depletion results in altered endosome maturation and disrupted lysosomal functions, leading to decreased EGFR degradation with its increased association with endosomes, which results in increased EGF-induced EGFR signaling, including AKT activation. Secondly, depletion of individual retromer-associated SNX proteins generally did not dissect out specific phenotypes associated with individual retromer subcomplexes. Global retromer function, therefore, is impacted indirectly when changes are made throughout its network of associated proteins. Alternatively, SNXs can act independently of retromer to influence EGFR trafficking. Finally, normal functionality of retromer and its SNXs is essential to ensure EGFR trafficking and signaling activation or termination occurs in a proper spatiotemporal manner.

## Data Availability

The HeLa cells (93021013; CCL-2) were originally obtained from Sigma-Aldrich, Castle Hill, NSW, Australia. Details of our generation of the various derived HeLa cell line models utilized in this study were described previously [14].
